# Directly measured free and total 25-hydroxyvitamin D levels in relation to metabolic health in multi-ethnic postmenopausal females in Saudi Arabia

**DOI:** 10.1530/EC-21-0445

**Published:** 2021-11-15

**Authors:** Shatha Alharazy, M Denise Robertson, Susan Lanham-New, Muhammad Imran Naseer, Adeel G Chaudhary, Eman Alissa

**Affiliations:** 1 Department of Physiology, Faculty of Medicine, King Abdulaziz University, Jeddah, Saudi Arabia; 2 Department of Nutritional Sciences, Faculty of Health and Medical Sciences, University of Surrey, Guildford, UK; 3 Centre of Excellence in Genomic Medicine Research, King Abdulaziz University, Jeddah, Saudi Arabia; 4 Department of Medical Laboratory Technology, Faculty of Applied Medical Sciences, King Abdulaziz University, Jeddah, Saudi Arabia; 5 Centre for Innovation in Personalized Medicine, King Abdulaziz University, Jeddah, Saudi Arabia; 6 Department of Clinical Biochemistry, Faculty of Medicine, King Abdulaziz University, Jeddah, Saudi Arabia

**Keywords:** postmenopausal, vitamin D, free 25-hydroxyvitamin D, free vitamin D, Saudi Arabia

## Abstract

**Background:**

Measurement of free 25-hydroyvitamin D (25(OH)D) status has been suggested as a more representative marker of vitamin D status than that of total 25(OH)D. Previously, free 25(OH)D could only be calculated indirectly; however, a newly developed direct assay for the measurement of free 25(OH)D is now available. The aim of this study therefore was to investigate directly measured total and free vitamin D levels association with metabolic health in postmenopausal healthy women living in Saudi Arabia.

**Methods:**

A sample of 302 postmenopausal women aged ≥50 years (*n*  = 302) living in Saudi Arabia were recruited in a cross-sectional study design. Blood samples were collected from subjects for measurement of serum levels of total 25(OH)D, directly measured free 25(OH)D, metabolic bone parameters, lipid profile, and other biochemical tests.

**Results:**

A positive correlation was found between directly measured free and total 25(OH)D (*r* = 0.64, *P<* 0.0001). Total but not free 25(OH)D showed significant association with serum intact parathyroid hormone (*P* = 0.004), whilst free 25(OH)D but not total 25(OH)D showed a significant association with total cholesterol and LDL-C (*P* = 0.032 and *P* = 0.045, respectively).

**Conclusions:**

Free 25(OH)D and total 25(OH)D were found to be consistently correlated but with different associations to metabolic health parameters. Further research is needed to determine which marker of vitamin D status would be the most appropriate in population studies.

## Introduction

Vitamin D plays an important role in numerous human metabolic functions including calcium (Ca) and phosphorus (PO_4_) hemostasis and bone growth and remodeling (1, 2). vitamin D stimulates Ca and PO_4_ absorption from the intestines, suppresses the production of parathyroid hormone (PTH), and enhances the renal tubular absorption of Ca and mobilization of Ca and PO_4_ from bone (3, 4, 5, 6). Vitamin D is not only vital for skeletal health but also involved in several extra-skeletal functions. Vitamin D deficiency has been associated with obesity, cardiometabolic risk, diabetes, age-related diseases, and autoimmune diseases (7, 8, 9, 10, 11). Additionally, there is emerging evidence that vitamin D sufficiency may be related to prevention of several pathological conditions including cardiovascular diseases, malignancies, autoimmune diseases, as well as severity of COVID19 (12, 13).

Vitamin D (in the form of vitamin D3) is primarily synthesized in the skin from a cholesterol derivative (7-dehydrocholeterol) reliant on UV-B radiation from exposure to the sun (14, 15, 16). Whether vitamin D is obtained from sunlight, from the diet, or through supplementation, 25-hydroxyvitamin D (25(OH)D) is considered as the major vitamin D metabolite that accurately represents vitamin D status (17, 18).

In healthy individuals, approximately 85–90% of vitamin D is bound to vitamin D-binding protein, with a low percentage (10–15%) being bound loosely to albumin, while the remainder (<1%, 0.03%) is said to be free (19). Based on the ‘free hormone hypothesis’, free 25(OH)D is the only form of 25(OH)D that is capable of cellular uptake and metabolism (20); hence, free 25(OH)D might be a more ‘appropriate surrogate marker’ for evaluating vitamin D status (21, 22). This hypothesis is confirmed by the finding that mice without vitamin D-binding protein who were expected to have deficient vitamin D levels subsequently did not show any indication of vitamin D deficiency but only when they were fed with vitamin D-deficient diet (23). It has been proposed that measuring total vitamin D might in fact be misleading in the assessment of vitamin D status in certain conditions such as liver and kidney diseases and that measuring free vitamin D might be more precise in evaluating vitamin D status in populations with more than one ethnic group (19, 24). For instance, it has been found that despite the lower total 25(OH)D levels in Black Americans in comparison with White Americans/Europeans, free 25(OH)D was not lower, thus explaining the stronger bone health found in Black individuals and adding credence to the hypothesis that free 25(OH)D might be superior to total in representing physiological vitamin D status (25, 26, 27). Free 25(OH)D can be measured either by means of a direct method, using a recently developed immunoassay, or estimated by calculation using the levels of albumin, vitamin D-binding protein and total 25(OH)D (20, 28). However, although commonly performed, the estimation of free 25(OH)D by the calculation method does not precisely represent the directly measured free 25(OH)D levels in several clinical situations (19).

Therefore, we aimed in this study to investigate total and directly measured free 25(OH)D association with metabolic health including bone metabolic parameters and lipid profile (primarily), lifestyle, and socio-demographic factors including ethnicity (secondarily). The study was conducted in a random cohort of multi-ethnic (white Middle Eastern and black African) postmenopausal women living in western Saudi Arabia where vitamin D deficiency is an extremely prevalent issue despite a high level of sunlight.

## Methods

### Subjects and study design

This is a cross-sectional study carried out in a sample of 302 postmenopausal women (age ≥50 years) in Jeddah, Saudi Arabia, (August 2018 to September 2019).

#### Sample size calculation

**Figure 1:**
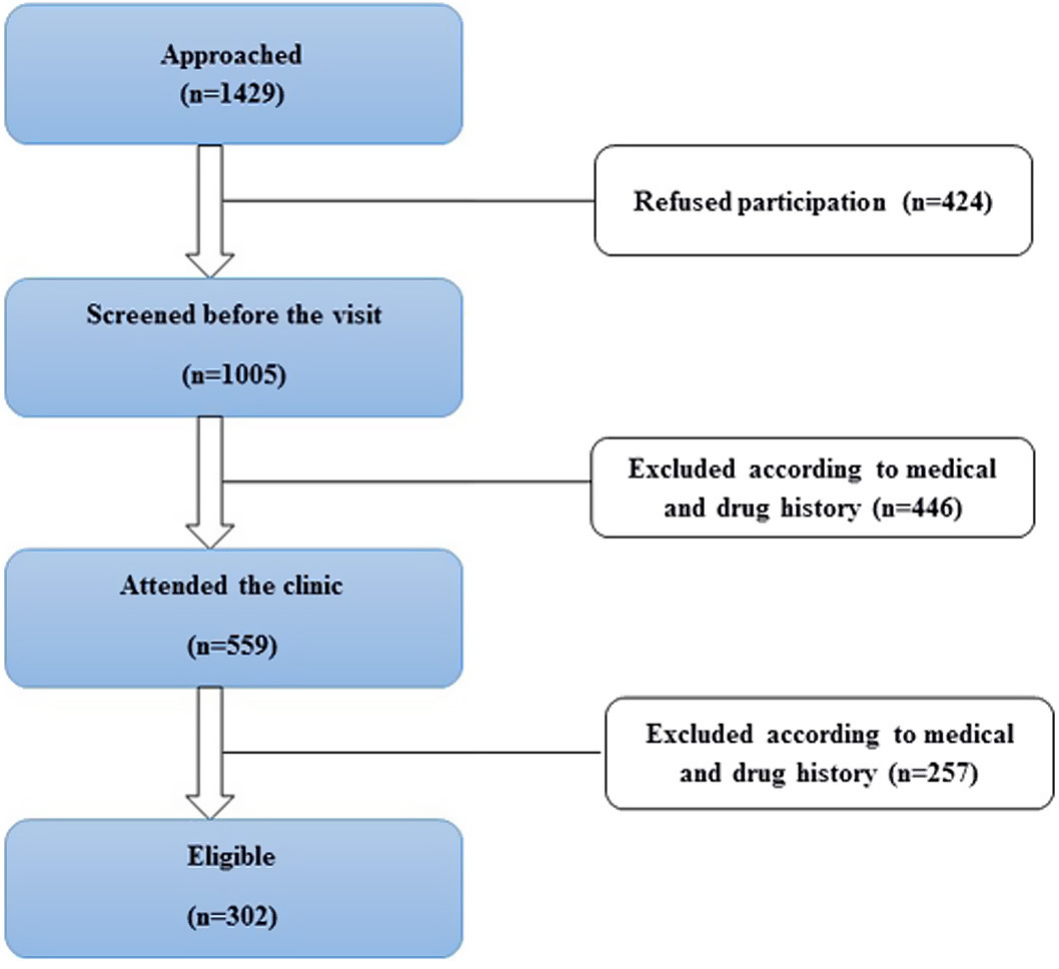
Flowchart of the study participants.

The total number of females of age range between 50 and 79 years in the western area of Saudi Arabia is approximately 430,739, according to the latest demographic survey of the general Saudi authority for statistics (https://www.stats.gov.sa/en) (29). The prevalence of vitamin D insufficiency (serum 25(OH)D <20 ng/mL; according to Institute of Medicine (IOM) (30) guidelines) among postmenopausal females in Jeddah (the western area of Saudi Arabia) is 80% (31). The maximum expected prevalence of serum vitamin D levels lower than the sufficient vitamin D level (<20 ng/mL) among those postmenopausal females is 90%. The level of confidence is 95% with a study power of 0.80. Accordingly, the calculated sample size was 240 females, as determined by a validated sample size determinant software program (Epi-Info, version 6, GA, USA). After several stages of exclusion (Fig. 1), a total of 302 women were collected for this study.

#### Recruitment

Recruitment of participating women for the current study was conducted from seven primary health care centers (PHCCs) scattered in Jeddah. Each PHCC represented one of the seven geographic divisions of Jeddah to ensure that the overall health condition of the participants will exemplify indiscriminately designated adult cohorts. We applied a multi-stage selection method. In the first stage, we designated a single PHCC representing each of the seven geographic divisions of Jeddah. In the second stage, files of the recorded female cohorts were selected by selecting randomly female samples from the designated PHCCs. In the third stage, all females in the nominated age group (50 years and above) were communicated for potential enrollment to the study with consideration of the defined inclusion criteria of this study. The sample size of randomly selected women from each center was proportionate to the number of the registered women in each center.

The study was undertaken at the Centre of Innovation in Personalized Medicine in King Fahad Medical Research Centre, King Abdulaziz University, Jeddah. A written well-informed consent for enrollment in the study was provided by each participant. Ethical standards of Declaration of Helsinki were followed throughout the study and ethical approval to conduct the study was obtained from the Unit of Biomedical Ethics, Center of Excellence in Genomic Medicine Research, King Abdulaziz University (05-CEGMR-Bioeth-2018).

Postmenopausal status for this study was defined as the ceasing of the menses for a period of 1 year and above, with a level of serum follicular-stimulating hormone greater than 15 IU/L. Exclusion criteria included history of chronic renal and liver disease, cancer, rheumatoid arthritis, malabsorption syndrome, hyperthyroidism, hyperparathyroidism, diabetes, or drug history including medications with potential influence on vitamin D levels (e.g. glucocorticoids, vitamin D supplements and anticonvulsants). Women with high levels of liver enzymes and creatinine were precluded (the normal clinical level of serum aspartate aminotransferase <45 U/L; alanine aminotransferase <50 U/L and alkaline phosphatase between 80 and 280 U/L; the normal level of serum creatinine in females <105 µmol/L). Women with thyroid-stimulating hormone levels below 0.465 mIU/L were also excluded.

#### Study protocol and data collection

Each participant completed a questionnaire (filled by the researcher) requesting data on medical history, drug history, menstrual history, socio-demographic (including ethnicity and skin tone), and lifestyle history (including sun exposure, veiling, dietary vitamin D intake, physical activity, and smoking habits).

Skin tone was noted for all participants relying on Fitzpatrick skin tone categorization (32). Use of sunscreen and extent (number of hours per week) of outdoor sunlight exposure in the past month was recorded for each participant. To evaluate parity, the total number of children was recorded for each participant. In consideration of the age group involved in the study, as well as the lifestyle of Saudi Arabia, a minimum of 30 min brisk walking (moderate aerobic activity) at least five times a week was considered as a cut-off for a reasonable form of exercise to be asked to the participants in the questionnaire (33).

In Saudi Arabia, the majority of women (particularly the elderly) wear cape and veil either for religious or cultural motives. Participating females covering their body and head while exposing their hands and face were regarded as partially covered, whereas females who covered their body and face while exposing hands and eyes were defined as totally covered.

Vitamin D dietary intake per day was assessed using semi-quantitative food frequency questionnaire (34). Food items included in the questionnaire included yogurt, milk, buttermilk, eggs, tuna, salmon, sardines, and beef liver as they are the most frequently consumed food rich in vitamin D in Saudi Arabia. Food frequency intake was presented as number of servings per day, week, or month. Vitamin D daily dietary intake in international units was then appraised to compare it with the estimated average requirement which is 600–800 IU/day according to the IOM reference for females aged 50 years and above (30).

### Blood collection and laboratory analysis

Blood samples were collected from each participant after an overnight fast. Serum free 25(OH)D was analyzed in duplicate by two steps immunoassay using ELISA kit (KAPF1991, Future Diagnostics Solutions B.V., Wijchen, Netherlands) with intra and inter-assay coefficient of variations (CVs) being between 5.3 and 6.4%. The first step of free 25(OH)D immunoassay was the incubation in which free 25(OH)D bound to the anti-vitamin D antibody was coated on the well of the microtiter plate. A specific amount of biotinylated 25(OH)D was added to each well after washing then the unbound biotinylated 25(OH)D was washed away and a streptavidin peroxidase conjugate was added. The second step was addition of chromogenic substrate. Lastly, the reaction was terminated by adding stop solution then absorbance (at 450 nm) is measured by a plate spectrophotometer.

Serum intact PTH and total 25(OH)D were quantified by direct competitive chemiluminescence immunoassay, using a LIASON auto-analyzer (DiaSorinInc, Stillwater, MN, USA) with intra and inter-assay CVs being 5.5 and 7.8% for serum total 25(OH)D and 4.9 and 4.5% for serum intact PTH, respectively. Vitamin D status was classified according to IOM guidelines (30): vitamin D sufficiency as 25(OH)D concentration range between 20 and 50 ng/mL or between 50 and 125 nmol/L, vitamin D insufficiency as the 25(OH)D concentration between 12 and 19 ng/mL or between 30 and 49nmol/L, and vitamin D deficiency as the 25(OH)D concentration <12 ng/mL or 30 nmol/L.

Serum albumin, creatinine, Ca, PO_4_, magnesium, lipid profile, blood glucose, liver enzymes (alanine aminotransferase, aspartate aminotransferase and alkaline phosphatase) were all measured using a VITROS 250 Clinical Chemistry auto-analyzer (Ortho-Clinical Diagnostics Inc., Rochester, NY, USA) with intra and inter-assay CVs less than 5%.

Serum LDL-C concentrations were assessed by calculations performed by the analyzer, using Friedewald equation (35) reliant on the obtained concentration of direct HDL-C, total cholesterol, and triglycerides. VLDL-C concentrations were calculated as the triglycerides level divided by 2.2.

Serum thyroid function test (free triiodothyronine, free thyroxin, and thyroid-stimulating hormone) and follicular-stimulating hormone levels were analyzed by immunoassays, using VITROS ECiQ (Ortho-Clinical Diagnostics Inc.) with intra and inter-assay CVs below 4%.

### Statistical analysis

SPSS program (v.25 SPSS Chicago Inc., 2011) was used for statistical analysis of data of this study. Kolmogorov–Smirnov test was used to test the normality of the data. All numerical parametric results were expressed as means ± s.d., while numerical non parametric results were presented as median (IQR). Descriptive results were expressed as a percentage of the total sample number. Spearman correlation was used to obtain associations between various parameters as data were not normally distributed. 25(OH)D levels were compared between groups using Kruskal–Wallis H test. *Post hoc* test (Dunn’s test) was utilized to determine which group was unlike from other. Statistical significance was determined at *P*-value of ≤0.05. Multiple stepwise regression analysis was conducted to find out potential predictors of vitamin D level as the dependent variable, with all independent variables that exhibited significant bivariate associations at *P*-value of ≤0.05.

## Results

The data concerning the general characteristics of the women taking part in this study are shown in Table 1. Serum levels of total and free 25(OH)D and screening biochemical parameters are shown in Tables 2 and  3.

**Table 1:** General characteristics of the participants. Numerical data are described as mean ± s.d. with normal distribution and as median (IQR) with non-normal distribution. Descriptive data are presented as *n* (%). % is percentage out of the total number of subjects.

Variables	Results (*n* = 459)				
	Median	IQR	Mean	s.d.	*n* (%)
Age (years)	58	53–63			
Age at menopause (years)	50	48–53			
Years since menopause	7	3–14			
Weight (kg)			75.2	16	
Height (cm)			154.3	6.5	
BMI (kg/m^2^)			31.5	7	
Waist circumference (cm)	90	84–99			
Hip circumference (cm)	110	102–119			
Waist-hip ratio	0.82	0.78–0.86			
Obesity classes*					
Normal (BMI: 18.5–24.9 kg/m^2^)					42 (14%)
Overweight (BMI: 25–29.9 kg/m^2^)					85 (28%)
Obese-class 1 (BMI: 30–34.9 kg/m^2^)					103 (34%)
Obese-class 2 (BMI: 35–39.9 kg/m^2^)					45 (15%)
Obese-class 3 (BMI: ≥40 kg/m^2^)					27 (9%)
Central obesity (Waist-hip ratio ≥0.8)					187 (62%)
Ethnicity^†^					
White (Arabic)					263 (87%)
Black (African)					28 (9%)
South Asian (Pakistani)					11 (4%)
Marital status					
Single					15 (5%)
Married					166 (55%)
Divorced					30 (10%)
Widow					91 (30%)
Education					
Illiterate					82 (27%)
Elementary					73 (24%)
Intermediate					45 (15%)
Secondary					54 (18%)
University					36 (12%)
Postgraduate					12 (4%)
Occupation					
Housewife					263 (87%)
Governmental employed					12 (4%)
Privately employed					3 (1%)
Retired					24 (8%)
Physical activity					
Yes					97 (32%)
No					205 (68%)
Smoking					
Yes					21 (7%)
No					281 (93%)
Hypertensive (according to their medical records)					
Yes					103 (34%)
No					199 (66%)
Use of sunscreen					0 (0%)
Dietary vitamin D intake (IU/day)	110	60–169			
Subjects consuming dietary vitamin D above estimated average requirement^‡^					0 (0%)
Vitamin D status^§^					
Optimal					121 (40%)
Insufficient					94 (31%)
Deficient					87 (29%)

*Obesity classifications are based on the World Health Organization obesity definitions (36, 37). ^†^Ethnicity was self-reported. ^‡^Estimated average requirement for women aged 50 years and over based on Institute of Medicine (IOM) recommendation (600–800 IU/day) (30). ^§^Vitamin D staus is classified according to 25(OH)D cut-off levels of IOM (30) (serum total 25(OH) <12 ng/mL deficiency, 12–19 ng/mL insufficiency, and 20–50 ng/mL optimal level).

**Table 2:** Serum levels of total and free 25(OH)D in overall and as classified by ethnicity. Difference between different ethnic groups were tested by Kruskal–Wallis H test.

Subjects	Total 25(OH)D (ng/mL)		Free 25(OH)D (pg/mL)		Percentage of free 25(OH)D out of total 25(OH)D (%)*	
	Median	IQR	Median	IQR	Median	IQR
Overall (*n* = 302)	17.6	10.6–24.4	4.32	3.29–5.72	0.026	0.018–0.033
White (*n* = 263)	18	11.25–25.3	4.33	3.31–5.79	0.026	0.019–0.033
Black (*n* = 28)	11.75	9.27–21.65	4.14	2.83–5.45	0.028	0.021–0.038
Asian (*n* = 11)	17.1	12.3–21.6	4.03	3.62–5.27	0.024	0.017–0.032
*P*-value for trend	0.131		0.658		0.588	

*Percentage of free 25(OH)D out of the total 25(OH)D was calculated by dividing free 25(OH)D levels in ng/mL over total 25(OH)D level in ng/mL then multiplied by 100.

25(OH)D, 25-hydroxyvitamin D.

**Table 3:** Biochemical characteristics of study subjects.

Variable	Results (*n* = 302)	
	Median	IQR
Serum intact PTH (pg/mL)	23.7	14.2–42
Serum albumin (g/L)	43	40–48
Serum Ca (mmol/L)	2.49	2.37–2.64
Serum corrected Ca (mmol/L)*	2.43	2.37–2.48
Serum PO_4_ (mmol/L)	1.36	1.24–1.51
Serum magnesium (mmol/L)	0.8	0.8–0.9
Fasting blood glucose (mmol/L)	5.6	5.2–6.2
Serum follicular-stimulating hormone (IU/L)	51.9	39–67.8
Serum thyroid-stimulating hormone (mIU/L)	2.37	1.39–3.7
Serum free triiodothyronine (ng/dL)	1.18	1.04–1.33
Serum free T3 (pg/mL)	3.72	3.26–4.13
Serum total cholesterol (mmol/L)	5.10	4.50–6
Serum triglyceride (mmol/L)	1.27	0.94–1.8
Serum HDL-C (mmol/L)	1.30	1.10–1.6
Serum LDL-C (mmol/L)	3.07	2.62–3.8
Serum VLDL-C (mmol/L)	0.58	0.43–0.83

*Corrected Ca was calculated as serum Ca + 0.02 (40−serum albumin).

25(OH)D, 25-hydroxyvitamin D; Ca, calcium; HDL-C, high density lipoprotein cholesterol; LDL-C, low density lipoprotein cholesterol; PO_4_, phosphate; PTH, parathyroid hormone; VLDL-C, very low density lipoprotein cholesterol.

Based on IOM vitamin D cut-off values (30), the participants were classified as 29% (*n*  = 87) with vitamin D deficiency (25(OH)D <12 ng/mL), 31% (*n*  = 94) with vitamin D insufficiency (25(OH)D level of 12–19 ng/mL), while 40% (*n*  = 121) had sufficient levels of vitamin D (25(OH)D level of 20–50 ng/mL). In our study population, total 25(OH)D and free 25(OH)D levels were not significantly associated with ethnicity (*P* = 0.13 and *P* = 0.66, respectively) (Table 2). In addition, they did not show any significant association with other sociodemographic factors (*P* > 0.05) (data not shown).

**Figure 2:**
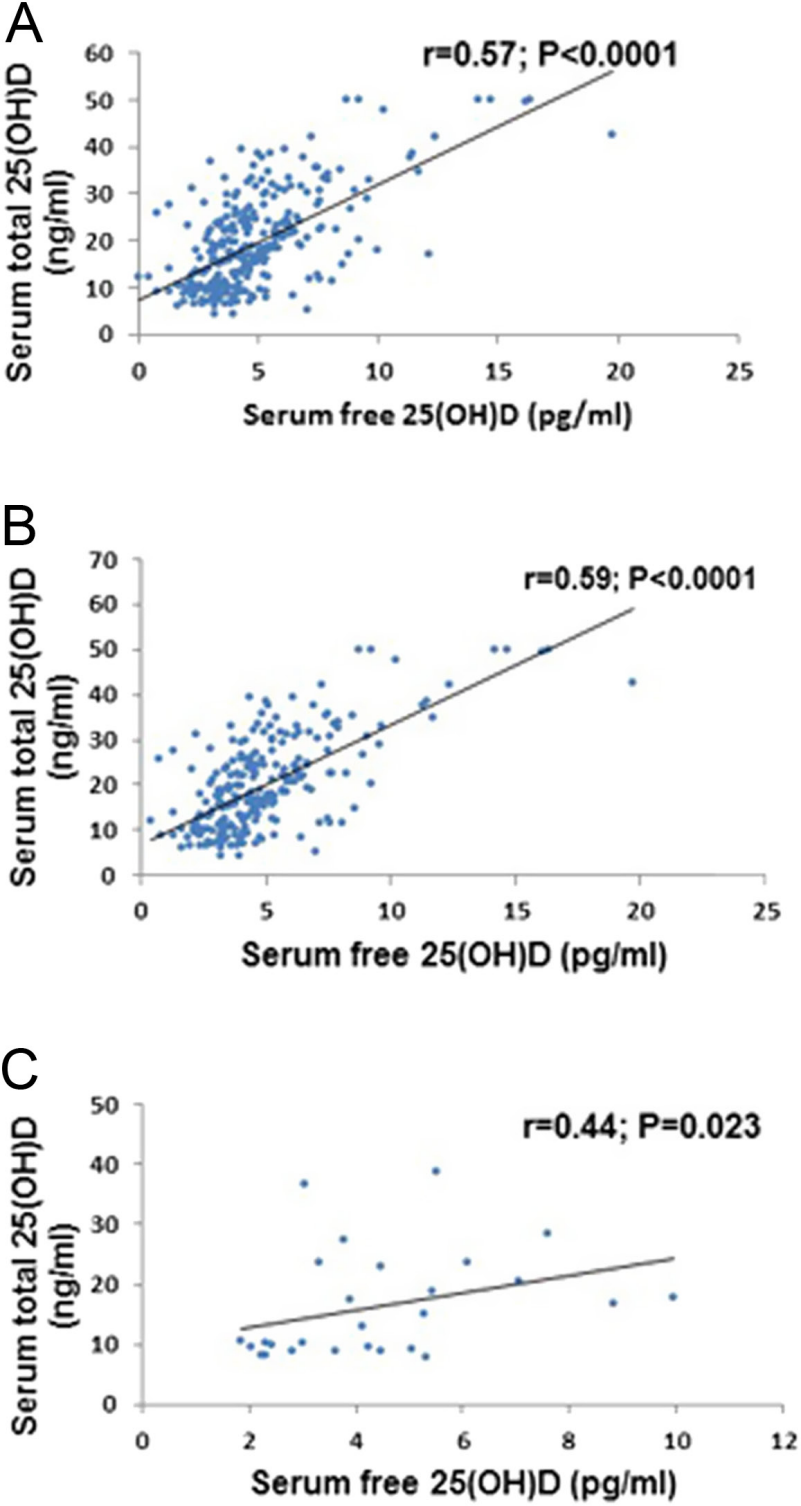
The relationship (Spearman’s correlation, two-tailed) between free 25(OH)D and total 25(OH)D. (A) In all study subjects (*n*  = 302). (B) In White subjects (*n*  = 263). (C) in Black subjects (*n*  = 28).

A significant correlation was found between free 25(OH)D and total 25(OH)D (*r* = 0.57, *P=* 0.001) (Fig. 2A). There was also a significant correlation between free 25(OH)D and total 25(OH)D among both White and Black groups (*r* = 0.59, *P=* 0.001 and *r* = 0.44, *P=* 0.023), respectively (Fig. 2B and  C). After stratification according to ethnicity, Asian race was excluded due to small frequency (only 11 participants).

**Figure 3:**
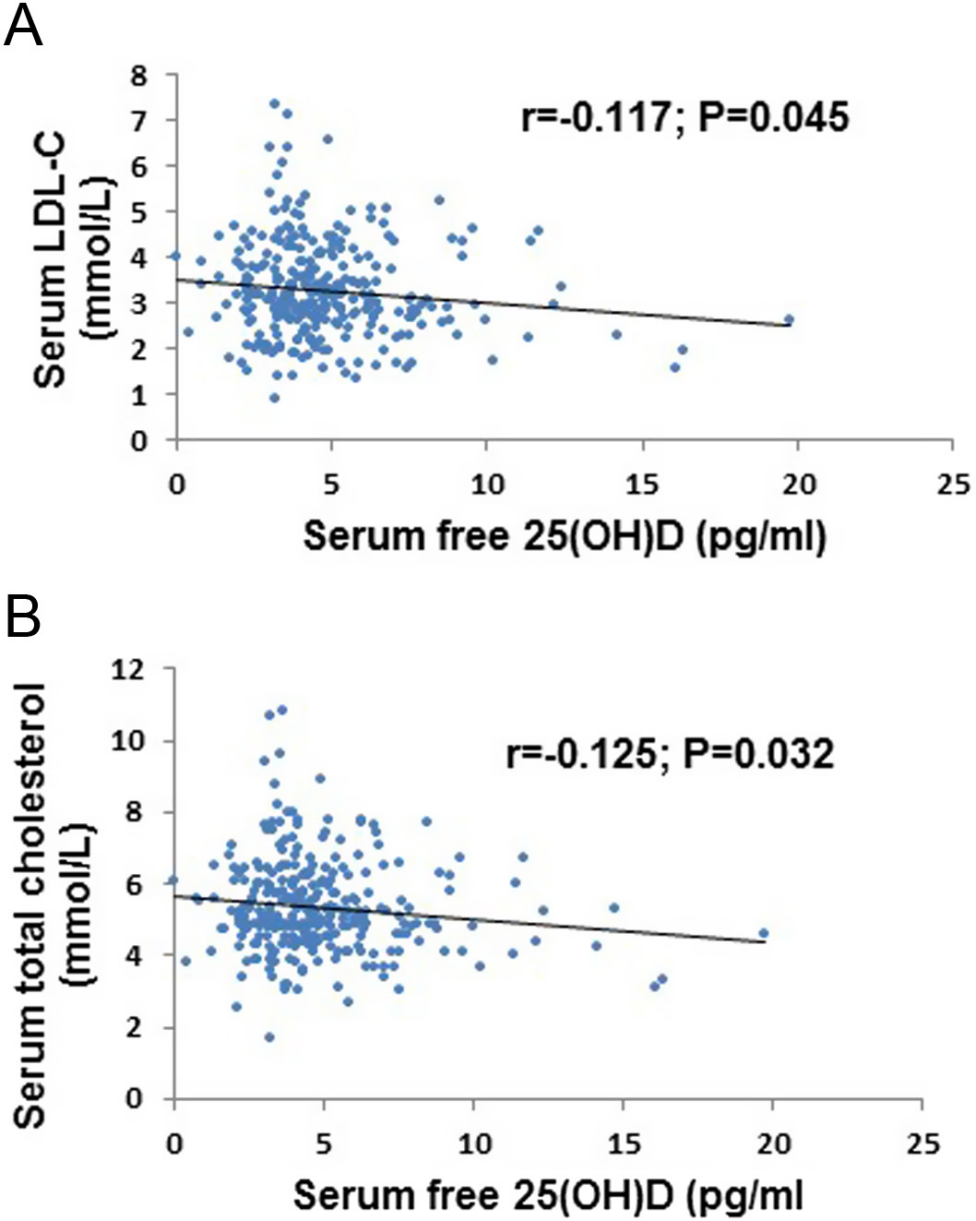
The significant relationship (Spearman’s correlation, two-tailed) between free 25(OH)D and lipid profile in all study subjects (*n*  = 302). (A) The relationship between free 25(OH)D and serum total cholesterol. (B) The relationship between free 25(OH)D and serum LDL-C.

Univariate analysis of total 25(OH)D and free 25(OH)D with bone-related parameters, anthropometric, and blood pressure parameters are shown in (Table 4). Total 25(OH)D in all studied women collectively was correlated inversely with intact PTH and positively with serum PO_4_ (*r* = −0.18, *P* = 0.004 and *r* = 0.13, *P* = 0.026, respectively). Similarly, total 25(O)D in White individuals was also inversely associated with serum intact PTH and positively with serum PO_4_ (*r* = −0.29, *P* = 0.001 and *r* = 0.14, *P* = 0.026, respectively) and serum albumin (*r* = 0.13, *P* = 0.04). Conversely, in Black individuals, no association was found between total 25(OH) and bone parameters. Importantly, no significant correlation was observed between free 25(OH)D level and bone parameters measured in this study. In addition, total and free 25(OH)D showed no association with BMI, waist circumference, hip circumference, waist-hip ratio, systolic blood pressure, or diastolic blood pressure in all participants collectively and in both White and Black ethnic groups. The data were tested to determine if there was any correlation between vitamin D status (total and free 25(OH)D) and lipid profile parameters including total cholesterol, triglyceride, HDL-C, LDL-C and VLDL-C. Total 25(OH)D was not associated with the lipid profile (Spearman’s two-tailed correlation: *P* = 0.71; *r* = −0.022 for total cholesterol, *P* = 0.085; *r* = −0.10 for triglyceride, *P* = 0.54; *r* = 0.036 for HDL-C, *P* = 0.95; *r* = −0.004 for LDL-C and *P* = 0.35; *r* = −0.056 for VLDL-C). On the other hand, free 25(OH)D was inversely associated with only total cholesterol and LDL-C (*P* = 0.032, *r* = −0.13 and *P* = 0.045, *r* = −0.12, respectively) (Fig. 3A and  B).

**Table 4:** Serum total and free 25(OH)D level association with anthropometric measures, blood pressure, and bone metabolic parameters. Total and free 25(OH)D association data are presented as *r* (*P*). All correlations were obtained by two-tailed Spearman’s correlation.

Variable	Overall (*n* = 302)		White ethnic group (*n* = 263)		Black ethnic group (*n* = 28)	
	Total 25(OH)D	Free 25(OH)D	Total 25(OH)D	Free 25(OH)D	Total 25(OH)D	Free 25(OH)D
BMI (kg/m^2^)	−0.057	−0.007	−0.043	−0.019	−0.001	0.10
	(0.33)	(0.90)	(0.49)	(0.76)	(0.10)	(0.62)
Waist circumference (cm)	−0.073	0.035	−0.12	0.004	0.25	0.21
	(0.22)	(0.56)	(0.055)	(0.95)	(0.21)	(0.32)
Hip circumference (cm)	−0.077	0.008	−0.094	−0.007	0.021	0.046
	(0.20)	(0.89)	(0.14)	(0.92)	(0.92)	(0.83)
Waist-hip ratio	0.009	0.04	−0.03	0.03	0.37	0.32
	(0.88)	(0.49)	(0.64)	(0.63)	(0.066)	(0.12)
Systolic blood pressure (mmHg)	−0.09	−0.031	−0.124	−0.095	−0.048	−0.30
	(0.16)	(0.63)	(0.07)	(0.16)	(0.82)	(0.15)
Diastolic blood pressure (mmHg)	−0.12	−0.041	−0.13	−0.062	0.12	−0.12
	(0.067)	(0.51)	(0.057)	(0.36)	(0.55)	(0.57)
Serum intact PTH (pg/mL)	−0.183*	−0.008	−0.285*	0.047	−0.16	−0.02
	(0.004)*	(−0.90)	(0.001)*	(0.49)	(0.41)	(0.92)
Serum albumin (g/L)	0.099	−0.086	0.13*	−0.052	−0.005	0.26
	(0.091)	(0.14)	(0.04)*	(0.41)	(0.98)	(0.20)
Serum Ca (mmol/L)	−0.006	−0.074	0.035	−0.072	−0.069	0.21
	(0.92)	(0.20)	(0.59)	(0.25)	(0.73)	(0.29)
Serum PO_4_ (mmol/L)	0.13*	0.078	0.14*	0.074	0.17	−0.099
	(0.026)*	(0.18)	(0.026)*	(0.26)	(0.39)	(0.62)
Serum magnesium (mmol/L)	−0.001	−0.10	0.052	−0.071	−0.08	−0.16
	(0.99)	(0.083)	(0.4)	(0.26)	(0.68)	(0.41)

*Significant correlation (*P* < 0.05).

25(OH)D, 25-hydroxyvitamin D; BMI, body mass index; Ca, calcium; PO_4_, phosphate; PTH is parathyroid hormone.

Total 25(OH)D and free 25(OH)D associations with skin tone, veiling type, parity, and sun exposure are shown in Table 5 for all subjects as whole. No significant differences were observed in total 25(OH)D in groups classified by skin tone, veiling type, parity, and sun exposure. In comparison, a significant free 25(OH)D difference was found between the groups of skin tone and veiling (*P* = 0.001 and *P* = 0.012, respectively) but not between parity or sun exposure groups (*P* = 0.17 and *P* = 0.32, respectively) (Table 5). *Post hoc* testing showed that free 25(OH)D in type II skin tone group is significantly different than in type III (*P* < 0.001), IV (*P* < 0.001), and V (*P* = 0.006). Additionally, the median free 25(OH)D was higher in the partially covered women than in the totally covered group.

**Table 5:** Differences in serum total and free 25(OH)D concentrations as classified by skin tone, veiling, parity, and sun exposure. 25(OH)D difference between different groups were tested by Kruskal–Wallis H test. % is percentage out of the total number of subjects.

Variables	*n* (%)	Total 25(OH)D ng/mL		Free 25(OH)D ng/mL	
		Median	IQR	Median	IQR
Skin tone (Fitzpatrick^†^)					
Type I (light, pale white)	3 (1%)	9.5	9–10	3.51	3.2–3.8
Type II (white, fair)	42 (14%)	20.45	10.6–31.7	5.6	4–7.6
Type III (medium white to olive)	97 (32%)	19.75	12.1–24.6	4.4	3.3–5.4
Type IV (olive, mid brown)	127 (42%)	16.6	11.5–24.3	4.1	3.3–5.2
Type V (brown, dark brown)	27 (9%)	10.95	9.4–19.7	3.7	2.8–5.3
Type VI (very dark brown, black)	6 (2%)	28.4	15.1–30.4	5.5	5.3–7.1
*P*-value for trend		0.055		0.001*	
Veiling type					
Totally covered, only eyes exposed	236 (78%)	17.1	10.4–23.8	4.1	3.2–5.5
Partially covered, face exposed	66 (22%)	19.85	11.8–26.9	4.7	4.0–6
*P*-value for trend		0.34		0.012*	
Parity					
No children	27 (9%)	16.4	11–22.5	3.6	2.9–4.5
1–2 children	30 (10%)	19	12.9–23.5	4.3	3.5–5.1
3–4 children	57 (19%)	17	10.7–24.9	4.1	3.3–6.7
5–6 children	97 (32%)	16.5	9.6–23.8	4.5	3.7–5.7
7+ children	91 (30%)	18.7	12–27.9	4.5	3.7–5.7
*P*-value for trend		0.48		0.17	
Sun exposure					
< 1 h/week	218 (72%)	17.7	10.7–24.3	4.3	3.3–5.5
1–2 h/week	42 (14%)	13.8	8.9–25.5	4.1	3.3–6.2
2–3 h/week	15 (5%)	13.8	10.2–20	4.7	3–5.5
>3 h/week	27 (9%)	20.7	13.5–24.6	5.3	3.5–7.1
*P*-value for trend		0.18		0.32	

**^†^** Fitzpatrick scale (25), *significant 25(OH)D difference (*P* < 0.05) between the groups.

IQR, inter-quartile range.

Multiple variables including anthropometric measures, blood pressure, skin tone, veiling, bone parameters, vitamin D supplementation, serum total cholesterol, and LDL-C level were tested by regression analysis. Multiple linear regression showing potential predictors of vitamin D status including total and free 25(OH)D are summarized in Table 6. Serum intact PTH and serum PO_4_ were statistically significant as predictors of serum total 25(OH)D levels, explaining 4.9% of the variance in serum total 25(OH)D levels. Alternatively, skin tone showed statistical significance as a predictor of free 25(OH)D status, contributing 2.2% of the variance in serum free 25(OH)D.

**Table 6:** Multiple regression analysis between serum 25(OH)D (free and total) level and independent variables.

Dependent variables	Independent variables	β	*P*	95% CI for β	
				Lower limit	Upper limit
Serum total 25(OH)D	Serum intact PTH (pg/mL)	−0.045	0.007*	−0.077	−0.012
	Serum PO4 (mmol/L)	5.644	0.046*	0.105	11.183
Total R^2^ = 0.049					
Serum free 25(OH)D	Skin tone	−0.409	0.01*	−0.719	−0.1
Total R^2^ = 0.022					

95% CI: confidence intervals; β, unstandardized regression coefficient; R^2^, percent variance explained by each variable. Variable inclusion with *P* < 0.05 and exclusion with *P* > 0.10.

*significant *P*-value (*P* < 0.05).

## Discussion

Free 25(OH)D (whether it is directly measured or calculated) was positively correlated with total 25(OH)D, which has been reported previously in other populations groups (19, 38, 39). Our data also support these findings. Regardless of the race or ethnic origin, total 25(OH)D in our study was associated significantly (*P* < 0.0001) with directly measured free 25(OH)D, which was consistent with observations found in United Kingdom and United States White individuals as well as African Americans and Gambians (39). However, when we investigated the association between total and free 25(OH)D with metabolic health parameters as well as socio-demographic and lifestyle factors, we found that free 25(OH)D did not behave entirely as total 25(OH)D. Free 25(OH)D (but not total) did not show an association with intact PTH; however, it did show an association with some parameters of lipid profile (total cholesterol and LDL-C), skin tone, and veiling which total 25(OH)D did not.

PTH, Ca, and PO_4_ are crucial markers related to bone health and bone metabolism. Serum intact PTH and PO_4_ were predictors for total 25(OH)D status in this study. The observed association between total 25(OH)D and PTH was not surprising as it has been previously extensively reported in the literature (38, 40, 41). In regards to the association between free 25(OH)D with bone-related parameters, our finding was in line with two studies (measuring directly free 25(OH)D) that reported lack of any significant association between free 25(OH)D and PTH (27, 42). However, it has been reported in several other studies that free 25(OH)D is inversely associated with PTH in a similar way to that of total 25(OH)D (38, 43, 44, 45, 46). These conflicting data could be attributed to several confounding factors affecting the relationship between vitamin D status and bone parameters such as age, sex, genetic variations, ethnicity, and even methodology used to measure total 25(OH)D. Reports on links between free 25(OH)D and bone markers are still conflicting and under debate. A very recent randomized-control trial in elderly (*n*  = 221) reporting an association of both free and total 25(OH)D with PTH and lack of association with Ca and bone turnover markers has concluded that no superiority of free 25(OH)D over total in evaluating markers of bone health (47). Overall, free 25(OH)D measurement in our results is not showing any superiority or advantage over the standard measurement of total vitamin D in respect to bone parameters. In fact, total 25(OH)D may be more appropriate in terms of detecting an association with bone metabolic parameters.

Raised PTH levels which are usually regarded as one of the indicators of vitamin D deficiency are more frequently seen in Black individuals than in White individuals (48). Additionally, the association between total 25(OH)D and PTH might be dissimilar in Black individuals and White individuals (49). In support with that, when our study subjects were categorized into White individuals and Black individuals, the previously observed associations between total 25(OH)D with bone parameters in all subjects collectively disappeared in Black individuals compared to White individuals that showed similar observed overall associations between vitamin D status and bone parameters. Our failure to find in Black individuals any association between vitamin D status and PTH levels was in agreement with what was found by Aloia *et al*. (27). This confirms the role of ethnicity in influencing vitamin D relationship with bone health.

Vitamin D deficiency has been linked with risk of cardiovascular diseases (50). Previous studies report a negative association between total 25(OH)D levels and cardiovascular risk markers and atherogenic lipid profile (51, 52). A review by Jorde *et al*. (52), comparing vitamin D status with lipid levels in 22 cross-sectional studies, found that total 25(OH)D was positively associated with LDL-C and HDL-C, positively or negatively associated with total cholesterol, and negatively associated with triglycerides. In contrast to this, our study did not find an association between total 25(OH)D and any of the measured lipid profile parameters. Free 25(OH)D, however, was inversely associated with serum total cholesterol and LDL-C levels (*r* = −0.125, *P* = 0.032 and *r* = −0.117, *P* = 0.045, respectively). Data on directly measured free 25(OH)D level and how it relates to lipid parameters is very limited in the literature. Most epidemiological studies relating vitamin D with cardiovascular risk are centered on total 25(OH)D only (53). Although our results of free 25(OH) relationship with lipid levels are inadequate to draw a definitive conclusion, free 25(OH)D exhibited a different relationship with lipids when compared with total 25(OH)D. However, whether future assessment of free 25(OH)D instead of total 25(OH)D can afford a new vision toward cardiovascular risk is still unclear. Additional research in this area is certainly needed.

Synthesis of vitamin D in the skin is influenced by many factors including age, skin coverage, the use of sunscreen, and skin pigmentation (54). People with darker skin tones tend to have lower circulating vitamin D levels as elevated skin melanin pigment hinders the penetration of UV sunrays and prevents consequent vitamin D dermal synthesis (55). In addition, housebound subjects and women who cover their body and/head while being outdoor for cultural or religious reasons, as the case in the participants of our study, are improbable to get sufficient vitamin D from sunrays (56, 57). Interestingly, free 25(OH)D in this study was associated with skin tone, with free 25(OH)D level being higher in the lighter type II skin tone group compared to the darker skin tone groups (skin type III, IV, and V). Supporting this, former studies found that vitamin D levels can vary according dermal melanin levels as melanin acts as a natural sunscreen (58). Individuals with darker skin color require longer duration of sun exposure to achieve similar vitamin D levels of those with lighter skin (59). It was shown by Armas *et al*. (55) that exposure to UV sunrays and skin type together form 80% of the differences in circulating serum levels of 25(OH)D resulted from UV sun exposure. However, the majority of studies investigating the vitamin D relationship with skin tone and sun exposure were focused on total vitamin D but not free. There is still a need to explore the free vitamin D relationship with skin tone and sun exposure. Veiling was also associated with free 25(OH)D but not with total 25(OH)D. Women who were partially covered with exposing their face and hands had higher serum free 25(OH)D concentrations than women who covered their body and face with exposing their eyes and hands only. Although veiling and sun exposure are consistently reported to be linked with vitamin D status (particularly total 25(OH)D) (1, 59, 60, 61), veiling association with total 25(OH)D was lacking in our study. However, results in women from United Arab Emarites (62) and Jordan (63) were parallel to our results. The unobserved differences in total vitamin D between subgroups of veiling or sun exposure found in our study are possibly due to the majority of participants being totally veiled and not being exposed adequately to sunlight. Furthermore, the overlap between veiling and sun exposure could be another reason as both partially (all covered except face and hands) and completely veiled (all covered except eyes and hands) women were exposing small skin areas and might not be different in aspects of adequate sun exposure and vitamin D synthesis. However, the exclusive free 25(OH)D association with veiling was conflicting and an explanation for this could not be drawn. This observation might suggest that free 25(OH)D can be influenced negatively by veiling more than total, which is confusing as there was no evidence of free 25(OH)D and sun exposure relationship. An expanded future study focusing on free and total 25(OH)D relation to sun exposure and veiling (with more details on sun exposure) might be essential to determine the influence of vitamin D sunlight-dermal synthesis on actual free vitamin D levels.

The current study has a number of strengths and limitations. Consideration of ethnicity when analyzing our vitamin D results is regarded as a point of strength. In addition, at the time that reports on actual free 25(OH)D data in general and in Saudi population particularly were inadequate. We measured free vitamin D directly instead of estimating its value by calculation as calculation methods seem to underestimate real free vitamin D. On the other hand, the current study has several limitations. First, the cross-sectional nature of the study design prevents us from determining causal relationships between vitamin D parameters and its correlates. Secondly, the present study relied on single measurements of vitamin D and did not consider the seasonal variation of vitamin D levels. However, the abundance of sunshine throughout the year in Saudi Arabia makes the seasonal aspect less relevant than in other international cohorts. Thirdly, the small sample size of Black and Asian ethnic groups, but this factor is not easily to be controlled as our study subjects were randomly selected according to their normal distribution in the Saudi community (in Jeddah city specifically). Lastly, this research used competitive chemiluminescence immunoassay to quantify vitamin D instead of using the gold standard method for measuring 25(OH), which is liquid chromatography tandem mass spectrometry.

## Conclusions

Free 25(OH)D association with total 25(OH) did not appear to be affected by ethnicity. Additionally, free 25(OH)D status did not exhibit an association with PTH compared to total 25(OH)D, but significant associations were seen with skin tone, veiling as well as total cholesterol and LDL-C. In conclusion, free 25(OH)D seemed not to follow total 25(OH)D in terms of association with metabolic health including bone metabolic parameters and lipid profile as well as with skin tone and veiling. More studies exploring further the role of actual free 25(OH)D in cardiovascular risk are needed as well as free 25(OH)D response to sun exposure and veiling. Moreover, additional studies are also needed to confirm the superiority of free 25(OH)D over total in the assessment of vitamin D status and its relationship to health outcomes.

## Declaration of interest

The authors declare that there is no conflict of interest that could be perceived as prejudicing the impartiality of the research reported.

## Funding

Joint supervision program, King Abdulaziz University http://dx.doi.org/10.13039/501100004054, Jeddah, Saudi Arabia. The funders had no role in study design, data collection and analysis, decision to publish, or preparation of the manuscript.

## Ethics approval and consent to participate

Ethical approval of this study was obtained from the Research Ethics Committee in Unit of Biomedical Ethics, Center of Excellence in Genomic Medicine Research, King Abdulaziz University (ref no. 05-CEGMR-Bioeth-2018). Fully informed, written consent was obtained from the participants.

## Availability of data and materials

The datasets used and/or analysed during the current study are available from the corresponding author on reasonable request.

## Author contribution statement

S A contributed to the study design and execution, data analysis and manuscript drafting. M D R and S L-N contributed to supervision, writing review and editing. M I N contributed to writing review. A C contributed to supervision. E A contributed to data analysis, writing review and supervision. All authors read and approved the final manuscript.
